# Acute Pancreatitis with Splenic Infarction as Early Postoperative Complication following Laparoscopic Sleeve Gastrectomy

**DOI:** 10.1155/2017/8398703

**Published:** 2017-04-13

**Authors:** Aleksandr Kalabin, Vishnu R. Mani, Ankita Mishra, Hector Depaz, Leaque Ahmed

**Affiliations:** ^1^Department of Surgery, Columbia University College of Physicians and Surgeons at Harlem Hospital Center, New York, NY 10037, USA; ^2^Department of Surgery, New York University School of Medicine, New York, NY 10016, USA

## Abstract

Obesity is becoming a global health burden along with its comorbidities. It imposes tremendous financial burden and health costs worldwide. Surgery has emerged as the definitive treatment option for morbidly obese patients with comorbidities. Laparoscopic sleeve gastrectomy is performed now more than ever making it imperative for physicians and surgeons to recognize both the common and the uncommon risks and complications associated with it. In this report we describe a rare early life-threatening postoperative complication following laparoscopic sleeve gastrectomy. From our extensive review of literature, there is no existing report of acute pancreatitis with splenic infarction postsleeve gastrectomy to this date.

## 1. Introduction

Obesity is a common medical condition in Western world that has tremendous negative effect on health with enormous economic burden. Greater than 400 million adults worldwide and more than one-third of US adults (approximately 78 million) are affected; moreover, its incidence is consistently trending upwards [1]. Obesity is defined as body-mass index (BMI) of over 30 kg/m^2^. Associated comorbidities such as heart disease, hypertension, diabetes mellitus type 2, obstructive sleep apnea, idiopathic intracranial hypertension, osteoarthritis, and thyroid disorders are just to name a few that are inseparably linked to obesity.

Laparoscopic sleeve gastrectomy (LSG) is a relatively new and effective restrictive surgery that helps obese people lose weight. LSG is being performed with increasing frequency worldwide and in USA. It is an effective treatment in achieving significant weight loss and improves most of the obesity related comorbidities [2]. Due to dramatic increase in the number of bariatric surgical procedures, surgeons in training and attending physicians should have ongoing understanding of common complications associated with LSG as well as recognize rare ones. Although the overall risks of major complication range between 2.9% and 12.9%, LSG is considered a relatively safe surgical option for weight loss [3]. Here we present a rare case of potentially life-threating complication following LSG: acute pancreatitis with splenic infarction in early postoperative period.

## 2. Case Report

20-year-old morbidly obese female (BMI = 43.3 kg/m^2^) with failed attempts to lose weight came to our bariatric clinic for weight reduction surgery. After full evaluation and discussion of bariatric surgical approaches, risks, and benefits related to surgery, the patient opted to proceed with LSG. As of note, no gallstones were noted during preoperative evaluation. The patient underwent uncomplicated LSG at our institution and was discharged home on postoperative day two. Patient was then followed up in bariatric clinic, reported of tolerating diet well and all laboratory tests were within normal limits. However, the patient returned to Emergency Room on postoperative day twelve with complaints of dull upper abdominal pain radiating to the back, nausea, and vomiting of both solids as well as liquids for the last twelve hours. On physical examination the patient had abdominal distension with epigastric and left upper-quadrant tenderness. Fever of 101.2°F and tachycardia of 106/min were noted. Laboratory data showed elevated WBC to 15.3 and serum Lipase up to 2432 U/L with subsequent increase in level up to 3384 U/L (*N* = 73–393). Hepatic function panel was normal. Ultrasonography was performed bedside and neither gall stones nor biliary tree dilatation was appreciated with poor visualization of pancreas secondary to bowel gas. Abdominal computer tomography scan with intravenous contrast showed two hypodense areas around medial aspect of spleen consistent with infarction and relatively normal appearing pancreas. There was absence of leakage from the staple line, fluid collections, or bowel obstruction. Patient was admitted to the surgical unit, closely monitored, and successfully managed conservatively with intravenous fluids, pain medications, antiemetics, and therapeutic anticoagulation with marked improvement of symptoms. Pain syndrome was reversed, the patient tolerated oral intake, and laboratory markers of pancreatic function skewed towards a downwards trend. The patient was discharged home with long-term anticoagulation on postoperative day 15 following LSG.

*LSG Technique*. In general, about 75%–80% of the greater curvature is excised, leaving a narrow stomach tube, also known as “banana.” The key phases of the procedures are (1) access or port placement: peritoneal cavity was entered using a 5 mm Optiview trocar in the midclavicular line below the left costal margin. This was done under direct vision using the optical trocar. A second 5 mm trocar was placed below the right costal margin at the midclavicular line. A third 5 mm trocar was placed in the anterior axillary line on the left side just below the costal margin. A 15 mm trocar was placed in the midline approximately 10 cm below the xiphoid. The Nathanson liver retractor was placed just below the xiphoid process and liver was retracted; (2) identification of distal point of transection of the stomach: the pylorus was identified using the prepyloric vein of Mayo as the marker. The greater curvature was measured approximately 5 cm from the pyloric region towards its mid portion; the lesser sac was entered by opening the gastrocolic ligament with LigaSure; (3) mobilization of greater curvature: this was accomplished by ultrasonic dissection of short gastric vessels and gastrosplenic and gastrocolic ligaments staying in the plane between the gastric wall and the gastroepiploic vessels, up to the angle of His. The adhesions between the angle of His and the diaphragm as well as those between the posterior gastric wall and the pancreas distally towards pylorus were separated using the LigaSure device ensuring no direct manipulation of the pancreas by retracting the gastric wall; (4) bougie insertion: a 38 French bougie was passed transorally into the pylorus and placed against the lesser curvature; (5) staple transection: a laparoscopic stapler was introduced and fired consecutively along the length of the bougie until the angle of His was reached; (6) staple line: it was reinforced. The specimen was removed through the 15 mm port; and (7) staple line inspection: it was done and drain placement was deemed not necessary [4].

## 3. Discussion

**Figure 1:**
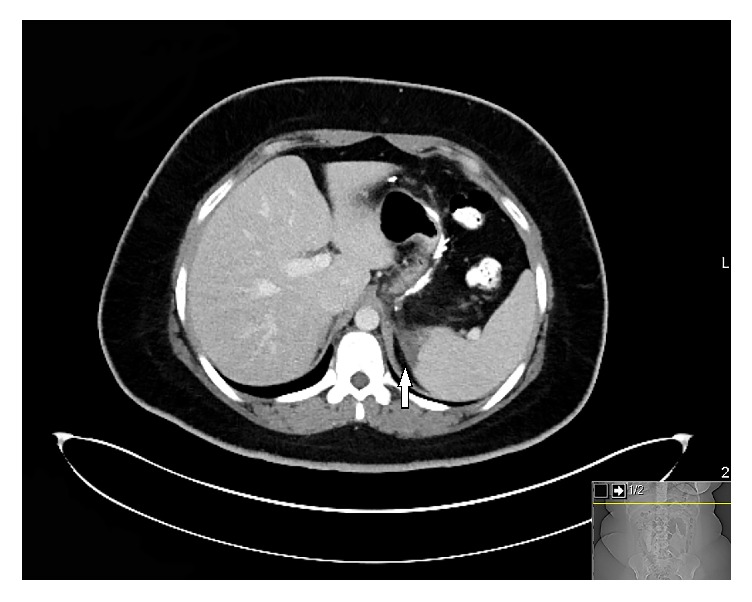
CT scan of abdomen depicting splenic infarction (arrow).

Although there is no consensus about an appropriate weight losing surgical procedure for obese patients, LSG, also known as “the sleeve,” is a relatively new procedure in bariatric surgeon's armamentarium [5]. Sleeve gastrectomy was originally described as part of biliopancreatic diversion with duodenal switch by Dr. Dough Hess (Bowling Green, Ohio, 1988) and later implemented as a laparoscopic stand-alone operation [6]. It is designed to reduce approximately 80 percent of the stomach with remaining pouch resembling a banana (Figure 1). After the surgery patients have a decreased appetite as they feel full with smaller meals consumed. Additionally LSG is associated with decreased secretion of ghrelin with resection of gastric fundus. Major advantages of LSG are volume restriction with preservation of pylorus and gastric function while preventing dumping, laparoscopic approach (even in patients who weigh over 500 lb), no foreign material required (as in gastric band placement), short hospital stay for one to two days, induction of rapid and significant weight loss, favorable changes in gut hormones that suppress hunger, reduction of appetite, and early satiety. The disadvantages are as follows: LSG is a nonreversible procedure and there is a potential for long-term vitamin deficiencies and higher early risk of stapling complications [7].

Complications could be classified on procedural (intraoperative) comprising bleeding, bowel perforation, diaphragmatic injury, injuries to other adjacent organs especially while dissecting attachments, and/or adhesions between the posterior surface of the stomach and the anterior pancreatic surface which could result in intraoperative pancreatic trauma and subsequent pancreatic disease including pancreatitis. Careful attention must be given towards the manipulation of short gastric vessels or distal splenic vessels, which could result in splenic infarction as well. Early complication (within two weeks) includes gastric leakage, hemorrhage, and abscess and rarely as in our case splenic infarction and acute pancreatitis. Delayed complication (after two weeks) includes stricture formation, nutritional deficiency, GERD, and port site hernia formation. On the other hand, the incidence of acute pancreatitis in early postoperative period following LSG is unknown in the literature to date. In one recently published cohort study only 28 patient (1.04%) out of 2695 following bariatric surgery developed acute pancreatitis during a median follow-up of 3.5 years [3] with rapid postoperative weight loss and the presence of gallstones as significant risk factors; however, this study does not specify exact time point or causation. Our case report links acute pancreatitis with splenic necrosis specifically to LSG as an early postoperative complication. A median period of 3.5 years for developing acute pancreatitis, in our opinion, poorly correlates this life-threating complication to the LSG itself. In another study only 138 of 3765 (3.6%) patients who underwent bariatric surgery developed postoperative pancreatic-biliary complications. Out of those 138 patients only 10 (0.27%) developed acute pancreatitis with mean time from surgery being 1.8 ± 1.4 years. Female gender, age > 50, cholelithiasis at the time of bariatric procedure, and Roux-en-Y gastric bypass were identified as predictive factors of pancreatic-biliary complications [8], with no mention of LSG as a definitive risk factor. On the other hand, portomesenteric vein thrombosis as an independent entity was found only in 5 patients out of 1236 (0.4%) following LSG in another recently published retrospective study [9] with smoking identified as a predominant risk factor.

We hypothesize that the development of acute pancreatitis with splenic infarction in patients undergoing LSG is secondary to manipulation of short gastric vessels and tributaries of the portal venous system along with combination of local inflammation. Peripheral (distal) splenic artery branches have poor collateral circulation and their injury during gastrectomy could result in an area of infarction distal to the involved branches. So accurate gastric mobilization during LSG is required to prevent any splenic vascular injury with concomitant complications. It is also imperative to take into consideration splenic pathology especially infarction, splenic parenchymal disease, and rupture being a rare but possible etiological contributor to pancreatitis although the reverse is more common. In one study published by Nores et al. in American Surgeon about the clinical spectrum of splenic infarction out of 59 patients 1 patient was complicated with pancreatitis. Acute pancreatitis with splenic infarction should be kept in mind while managing patients following LSG with complaints of abdominal pain. Aforementioned life-threatening complication probably could be prevented with meticulous tissue dissection and surgeon's experience during stomach mobilization paying careful attention to pancreas.

## 4. Conclusion

As previously stated the incidence of obesity is on the rise and more patients are undergoing bariatric surgical procedures, LSG being the popular choice. This fact is correlated with the escalating incidence of complications associated with the procedure and it is quintessential for surgeons to be aware of potential complications and to be able to diagnose and manage them. The purpose of presenting our case is to shed light on rare but possible life-threatening complication following LSG. As the number of LSG performed annually continues to increase, a high index of suspicion should remain for acute pancreatitis with possible portomesenteric thrombosis and subsequent splenic infarction or vice versa in patients presenting with abdominal pain after LSG. Additionally, surgeons should keep in mind that real incidence could be much higher because many cases of splenic infarct could be “silent” due to unspecific clinical picture [10]. In our opinion missed diagnosis of acute pancreatitis with splenic infarction could be a potential cause of abscess formation or secondary leakage increasing morbidity and deserves particular attention. It should certainly be considered in differential diagnosis of patients presenting to the emergency department due to abdominal pain, especially in view of previous LSG.
